# Elucidating the Mechanism of Absorption of Fast-Acting Insulin Aspart: The Role of Niacinamide

**DOI:** 10.1007/s11095-019-2578-7

**Published:** 2019-02-11

**Authors:** Jonas Kildegaard, Stephen T. Buckley, Rasmus H. Nielsen, Gro K. Povlsen, Torben Seested, Ulla Ribel, Helle B. Olsen, Svend Ludvigsen, Claus B. Jeppesen, Hanne H. F. Refsgaard, Kristian M. Bendtsen, Niels R. Kristensen, Susanne Hostrup, Jeppe Sturis

**Affiliations:** Novo Nordisk A/S, Novo Nordisk Park, DK-2760 Måløv, Denmark

**Keywords:** absorption; fast-acting insulin aspart; niacinamide; oligomerization; vasodilation

## Abstract

**Purpose:**

Fast-acting insulin aspart (faster aspart) is a novel formulation of insulin aspart containing two additional excipients: niacinamide, to increase early absorption, and L-arginine, to optimize stability. The aim of this study was to evaluate the impact of niacinamide on insulin aspart absorption and to investigate the mechanism of action underlying the accelerated absorption.

**Methods:**

The impact of niacinamide was assessed in pharmacokinetic analyses in pigs and humans, small angle X-ray scattering experiments, trans-endothelial transport assays, vascular tension measurements, and subcutaneous blood flow imaging.

**Results:**

Niacinamide increased the rate of early insulin aspart absorption in pigs, and pharmacokinetic modelling revealed this effect to be most pronounced up to ~30–40 min after injection in humans. Niacinamide increased the relative monomer fraction of insulin aspart by ~35%, and the apparent permeability of insulin aspart across an endothelial cell barrier by ~27%. Niacinamide also induced a concentration-dependent vasorelaxation of porcine arteries, and increased skin perfusion in pigs.

**Conclusion:**

Niacinamide mediates the acceleration of initial insulin aspart absorption, and the mechanism of action appears to be multifaceted. Niacinamide increases the initial abundance of insulin aspart monomers and transport of insulin aspart after subcutaneous administration, and also mediates a transient, local vasodilatory effect.

**Electronic supplementary material:**

The online version of this article (10.1007/s11095-019-2578-7) contains supplementary material, which is available to authorized users.

## Introduction

Limiting excessive postprandial glucose (PPG) excursions is a major challenge in the treatment of diabetes. Postprandial hyperglycemia contributes to overall glycemia, as determined by glycated hemoglobin (HbA_1c_), and has been proposed as an independent risk factor for cardiovascular disease (1). Bolus insulin therapy aims to mimic the normal mealtime insulin response to reduce postprandial hyperglycemia while avoiding hypoglycemia. Current rapid-acting insulin analogues, such as insulin aspart, have been developed to have faster absorption profiles and provide an earlier onset of action than regular human insulin (RHI) (2). Insulin aspart is identical to RHI, with the exception of a single amino acid replacement, resulting in charge repulsion and a more rapid dissociation of insulin hexamers into dimers and monomers (3,4). By reducing the tendency to remain self-associated as hexamers after subcutaneous administration, the speed of absorption into the circulation is increased, and insulin aspart reaches peak plasma concentrations more rapidly compared with RHI, to better mimic the normal mealtime insulin response (5–7).

Nevertheless, despite such advances in insulin development, many patients with type 1 diabetes (T1D) or insulin-dependent type 2 diabetes (T2D) remain unable to control postprandial hyperglycemia and hence meet or maintain HbA_1c_ targets. An unmet need therefore exists for a mealtime bolus insulin with even faster absorption (2). Fast-acting insulin aspart (faster aspart) is a novel formulation of insulin aspart containing two excipients: niacinamide (170 mM) and L-arginine (20 mM) (8). The product provides an earlier onset of appearance, a greater early glucose-lowering effect, and a shorter duration of action compared with insulin aspart (9). In clinical trials in patients with T1D and T2D, faster aspart demonstrated non-inferiority to insulin aspart with respect to HbA_1c_ reduction, with superior PPG control and no increased hypoglycemia risk (10–12).

L-arginine was selected as an excipient to optimize the stability of the formulation, and niacinamide was selected to increase the early absorption of insulin aspart (13). The oligomerization of insulin is partly driven by hydrophobic interactions, as the thermodynamically favorable shielding of hydrophobic residues plays an important role in formation of the insulin hexamer. Niacinamide and many of its derivatives belong to a group of compounds classified as hydrotropes (14–17). Hydrotropic compounds have the ability to increase the solubility of poorly soluble substances in aqueous solution without forming a separate hydrophobic phase (18). At high concentrations, a hydrotrope such as niacinamide is considered likely to shift the balance of oligomerization of insulin from hexamers towards dimers and monomers, making the readily absorbed monomer more abundant.

Additionally, niacinamide acts as a vasodilator (19–21), and a possible increase in local blood flow at the injection site may also augment insulin absorption. Indeed, the vasodilator prostaglandin E1 has been shown to increase early absorption of human insulin in patients with diabetes (22), and infusion-site warming to increase local blood flow improves the pharmacokinetic (PK) profile of insulin analogues and lowers PPG excursions in patients with T1D using insulin pump therapy (23).

The aim of this study was to further evaluate the impact of niacinamide on insulin aspart absorption kinetics and to investigate whether the accelerated absorption facilitated by niacinamide could be the result of decreased insulin aspart self-association, increased blood perfusion of the depot or both.

## Materials and Methods

### Studies Using Pigs

#### Compliance with Ethical Standards

Animal experiments were carried out in accordance with the Danish Act on Experiments on Animals, the Appendix A of ETS 123 and EU Directive 2010/63. A procedure project license was issued by the national authority. All animal experiments were approved by the Novo Nordisk Animal Welfare and Ethical Review Body before submission to the national authority.

#### Housing and Husbandry

Domestic pigs (Landrace-Yorkshire-Duroc [LYD]) were accommodated and cared for by competent personnel and supervised by laboratory animal veterinarians. Animals were housed with 12-h day–night cycles with lights on at 6:00 am, in a holding room with target temperatures of 21±2 °C and target relative humidity from 30–70%. Pigs were single housed in enriched pens with the possibility of social interaction. They had free access to water and were fed a standard diet according to their weight. Pens contained wood shavings, straw and toys for environmental enrichment. Pigs were allowed daily exercise in corridors and received apples as treats.

### Pharmacokinetic Studies in Pigs to Assess the Effect of Niacinamide on Insulin Aspart Absorption

Formulations of insulin aspart with niacinamide were administered subcutaneously to female pigs (55–85 kg) in a full cross-over dose-response study. The composition of the formulations are shown in Table I. Formulations with 1-methyl-niacinamide, an analogue of niacinamide, were also administered. Plasma samples were obtained from the pigs for 360 min following administration. One pig was replaced after the third day of dosing due to pneumonia.

**Table I:** Composition of Formulations Administered Subcutaneously to Pigs

Insulin aspart (mM)	Zinc acetate (mM)	Phenol (mM)	m-cresol (mM)	Sodium chloride (mM)	Glycerol % (w/vol)	Niacinamide (mM)	pH
0.6	0.3	16	16	10	1.6	-	7.4
0.6	0.3	16	16	10	1.6	8	7.4
0.6	0.3	16	16	10	1.4	40	7.4
0.6	0.3	16	16	10	0.9	130	7.4

Plasma samples were analyzed using a Luminescent Oxygen Channeling Immunoassay (LOCI or AlphaLISA) (24). The assay was performed by incubating samples with donor beads coated with streptavidin, acceptor beads conjugated with a monoclonal antibody (HUI-018) directed against human insulin and a second biotinylated monoclonal antibody (X14-6-F34) directed against insulin aspart. A light signal generated from a chemiluminescent reaction within the bead–aggregate–immune complex was measured and used to quantify the amount of insulin aspart in each sample.

The rate of absorption for each formulation was expressed as the area under the curve (AUC)_0-15_/AUC_0-60_ ratio. Ratios were compared using unpaired t-tests.

### Tissue Sampling in Pigs to Evaluate the Kinetics of Subcutaneous Dispersion and Disappearance of Niacinamide

Pigs (70–90 kg) were anesthetized using Zoletil mixture (1 mg/10 kg; Virbac, Denmark) with intravenous supplement of propolipid (Fresenius Kabi, Sweden). Pigs were placed on their side and injected in the neck region with 50 μl of phosphate buffer (10 mM; pH 7.4) supplemented with niacinamide (170 mM; adjusted to isotonic with 85 mM NaCl) or 1-methyl-niacinamide (170 mM). Tissue samples measuring ~2 x 2 cm were excised at 0 min (acute), 2, 5, 15 or 60 min after injection and immediately frozen in 2-methyl-butane. To evaluate whether niacinamide and 1-methyl-niacinamide were entirely confined within the excised tissue, 1 cm thick sections along the four edges of the 0 min (acute) sample were also excised. Following euthanasia by overdose of pentobarbital (Lundbeck, Denmark), tissue samples measuring ~10 x 10 cm were excised and injected to obtain *ex vivo* 0 min (acute) and 60 min depots.

#### Tissue Homogenization

Tissue samples were cut into smaller blocks, thawed and diluted with phosphate buffer (180 mM; pH 7.4) at a ratio of 3 ml to 1 g of tissue. Tissue was homogenized with two cycles (one cycle consisting of 10 sec homogenization, 60 sec pause, 10 sec homogenization) at 8800 rpm (Precellys Evolution homogenizer, Bertin Instruments, France) or until the sample was considered homogenized.

#### Liquid Chromatography–Mass Spectrometry (LC-MS/MS) Quantification

Protein was precipitated from the tissue homogenate by adding three volumes of acetonitrile. Samples were centrifuged for 30 min at 4°C and the supernatants analyzed on a QTRAP^®^ Triple Quadrupole coupled with an Acquity liquid chromatography system (Waters, USA). Quantification was performed from a calibration curve prepared by spiking buffer with niacinamide or 1-methyl-niacinamide over a concentration range of 1 to 1000 ng/ml.

### PK Modelling Using Available Data from Human Patients with Diabetes

Data from previously published PK studies in human patients with T1D (25,26) were used to estimate the impact of niacinamide on the rate of insulin aspart absorption relative to the amount of insulin aspart in the subcutaneous depot. Two different modelling methods were utilized: one using a deconvolution approach and a second using a population modelling approach (described in detail in the Supplementary Material).

### X-ray Scattering to Assess the Influence of Niacinamide on Insulin Aspart Oligomerization

#### Small Angle X-Ray Scattering (SAXS) Sample Preparation

Samples of insulin aspart, containing phenol, m-cresol and niacinamide, were prepared to reflect the pharmaceutical formulation of faster aspart (Table II). Similar samples without the excipients were also prepared. To emulate conditions after subcutaneous injection, samples were diluted 1:1 with Hank's Balanced Salt Solution (HBSS) buffer supplemented with 4-(2-hydroxyethyl)-1-piperazineethanesulfonic acid (HEPES) (10 mM; pH 7.4). Ovalbumin, glycerol and Tween 20 (included in the HBSS buffer used for the trans-endothelial transport assay) were omitted as these compounds gave rise to substantial X-ray scattering.

**Table II:** Composition of Insulin Aspart Samples

Sample	Insulin aspart (mM)	Zinc acetate (mM)	Phenol (mM)	m-cresol (mM)	Arginine hydrochloride (mM)	Niacinamide (mM)	Phosphate (mM)	pH
1	0.6	0.3	16	16	20	170	3	7.1
2	0.6	0.3	16	16	20	-	3	7.1
3	0.6	0.3	-	-	20	170	3	7.1
4	0.6	0.3	-	-	20	-	3	7.1

Samples were diluted 1:1 in HBSS buffer prior to the SAXS measurements. Samples used for background subtraction were identical, but did not contain insulin aspart. Samples containing 1-methyl-niacinamide, a non-functional niacinamide analogue, were used as a negative control. HBSS, Hank's Balanced Salt Solution; SAXS, small angle X-ray scattering

To emulate Zn^2+^-free subcutaneous conditions, insulin aspart samples were also prepared in phosphate buffered saline (PBS) buffer (140 mM NaCl, 10 mM phosphate; pH 7.4) and interstitial fluid-like (ISF) buffer ([in mM]: 140 NaCl, 4 KCl, 2 CaCl_2_, 1 MgSO_4_, 10 phosphate; pH 7.4) with niacinamide (0–380 mM) or 1-methyl-niacinamide.

#### SAXS Data Collection and Analysis

SAXS measurements were made using a BioSAXS-1000 Rigarku instrument that features a two-dimensional Kratky design with focus on the detector to eliminate smearing. The scattering signal was recorded with a Pilatus 100K Dectris detector covering the scattering vector range (q=4*π*sin*θ*/*λ*) from 0.008Å^-1^ to 0.658Å^-1^, corresponding to real space distances of 9.5 to 785Å. An automatic sample changer was used for sample and buffer handling. Samples were kept at 8^o^C in the autosampler prior to the measurements. Twelve two-dimensional frames were recorded using 10 min exposures. An average was taken after visual inspection and comparison of data quality of the individual subframes. Q-calibration of the instrument was carried out with silver behenate and radial averaging was performed with Rigaku’s instrument software. Averaging and buffer subtraction were conducted using Primus.

The quality of the recorded SAXS data was reasonable, considering the relatively low sample concentration (1.75 mg/ml) (Supplementary Fig. S1). The computer program Shanum (27) was employed to estimate the data content in terms of number of useful Shannon channels. The number of Shannon channels ranged from 10 to 16, with an average of 13. No signs of aggregation or precipitation were observed. Moreover, as would be expected from the relatively low protein concentration and moderate ionic strength of the solvent (0.3 mM insulin aspart), very little distortion of the low q-region due to inter-particle interference effects was observed.

The SAXS data could be satisfactorily described using a model consisting of monomers, dimers and hexamers. However, the estimated volume fractions of dimers were in all cases negligible and, therefore, this model was discarded in favor of a simpler monomer-hexamer model, which, due to the lower number of fitting parameters, resulted in slightly improved reduced chi-squared statistic. Theoretical calculated form factors of monomers and hexamers were obtained using Crysol based on the crystal structures of 1MSO and 1EV6, respectively. Subsequently, the calculated form factors were fitted to the experimental data with the program Oligomer. The programs Primus, Crysol and Oligomer are part of the ATSAS package (28). To investigate the effect of niacinamide on oligomer number, a model-independent approach was applied where the weighted average molecular mass was calculated based on forward scattering (i.e. the scattering intensity extrapolated to zero intensity). Subsequently, the average oligomer number was obtained by dividing the molecular mass with the formula weight of the molecule.

### *In vitro* Assessment of Transport of Insulin Aspart Across an Endothelial Cell Barrier

#### Cell Culture

Primary human dermal microvascular endothelial cell (HDMEC; PromoCell GmBH, Germany) cultures were grown in Endothelial Cell Growth Medium supplemented with 5% fetal bovine serum, human epidermal growth factor, hydrocortisone, gentamicin (30 mg/ml), amphotericin (15 μg/ml), vascular endothelial growth factor, human basic fibroblast growth factor, R3-insulin-like growth factor (IGF)-1 and ascorbic acid. HDMEC cells were seeded at a density of 1.2×10^5^ cells/well onto polyester filters in 24-well Transwell^®^ plates (0.3 cm^2^, 0.4 μm pore size) pre-coated with fibronectin. Cells were cultured at 37°C in 5% CO_2_, and culture medium was exchanged every other day. Experiments were performed after 5–6 days in culture.

#### Trans-Endothelial Transport Assay

Cells were allowed to equilibrate for 30 min in HBSS buffer supplemented with HEPES (10 mM), 0.1% ovalbumin and 0.005% Tween 20 at pH 7.4. Following removal of the buffer, human insulin and insulin aspart samples (see Table II for composition) with niacinamide or 1-methyl-niacinamide were diluted 1:1 with HBSS buffer and added to the cell monolayers. Donor samples were collected at 0 min and 60 min (end of experiment), and receiver samples were collected at 5, 10, 15, 30, 45 and 60 min. The study was performed at 37°C in 5% CO_2_, with shaking at 30 rpm. The amount of human insulin or insulin aspart in the samples was determined by luminescent oxygen channeling assay. Both donor and receiver samples were diluted prior to analysis.

#### Data Analysis

The amount of compound transported from the donor chamber to the receiver chamber was calculated as J=ΔQ/Δt/A, where *ΔQ/Δt* is the accumulated amount of intact human insulin or insulin aspart on the receiver side and *A* is the area of the cell monolayer. The apparent permeability (*P*_*app*_) was determined by linear regression according to Fick’s first law: *P*_*app*_=J/C_0_, where *C*_*0*_ is the initial concentration on the donor side. The ratio ‘amount accumulated_0-15min_/amount accumulated_0-60min_’ reflects the amount of insulin aspart accumulated in the receiver chamber over the first 15 min of the study, expressed as a percentage of the amount accumulated over the entire study. Where applicable, values were normalized to account for differences in the start concentration measured in the donor chamber.

### *Ex Vivo* Assessment of the Effect of Niacinamide on the Vasorelaxation of Porcine Arteries

Biopsies of skin and subcutaneous tissue (~3 cm^2^) were taken from the gluteal region of female pigs (50–80 kg). Arteries with internal luminal diameters of 100–700 μm were dissected from subcutaneous fat approximately 5 mm below the skin surface and placed in ice-cold physiological saline solution (PSS) composed of (in mM): 119 NaCl, 4.7 KCl, 2.5 CaCl_2_, 15 NaHCO_3_, 1.2 MgSO_4_, 1.2 KH_2_PO_4_, 11 glucose and 0.03 ethylenediaminetetraacetic acid (EDTA). Vessels were cut into 1.5 mm cylindrical segments, threaded onto two 40 μm diameter stainless steel wires and mounted on a Mulvany-Halpern myograph (Danish Myograph Technology, A/S). Vessel segments were immersed in PSS at 37°C and continuously aerated with 5% CO_2_/95% O_2_ to maintain a stable pH of 7.4. Contractile force was recorded via a PowerLab unit and Labchart software (AD Instruments).

Vessels were pre-stretched in calcium-free PSS to a lumen diameter of 0.9 × L100, where L100 is an estimate of the diameter of the vessel under a passive transmural pressure of 100 mmHg. This was performed using the normalization module in LabChart7. Following pre-stretch, vessels were equilibrated in PSS for minimum 30 min, after which three potassium-induced constrictions were performed using an isotonic solution containing 63 mm K^+^, obtained by partial substitution of KCl for NaCl in PSS. Vessels were then pre-constricted with U46619 (1 μM; a thromboxane receptor agonist) and allowed to stabilize in the pre-constricted state for 10 min, after which niacinamide or 1-methyl-niacinamide was added in cumulatively increasing concentrations.

### *In Vivo* Assessments of the Effect of Niacinamide on Regional Subcutaneous Blood Flow in Pigs

#### Xenon-133 (Xe-133) Washout

Xe-133 was dissolved in niacinamide (170 mM), prostaglandin E1 (4 μg/ml) and saline vehicles, each with and without insulin aspart, and injected into the neck of pigs (60–100 kg). For each experiment, an insulin aspart containing vehicle was injected on one side of a pig, while the same vehicle without insulin aspart was injected into the other side. Prostaglandin E1 was included as a positive control. A gamma sensor was mounted over each injection site to measure the decay in Xe-133 signal. The change in signal during the first 15 min for each of the three vehicles (without insulin aspart) was analyzed using linear regression to give regression line slopes (in % per min) for each animal. Regression line slopes in the 0–15 min interval were compared using one-way analysis of variance (ANOVA).

#### Laser Speckle Contrast Analysis (LASCA)

Formulations of prostaglandin E1 (10 μg/ml), niacinamide (170 mM) or saline were injected intradermally in the neck area of anesthetized pigs. A PeriCam PSI system (Perimed, Sweden) was used to record one image per second for approximately 15 min. Perfusion units per min were compared using one-way ANOVA.

#### Laser Doppler Imaging (LDI)

LDI (moorLDI2-IR, Moor Instruments, UK) was used to record a series of images (approximately one image per 3.5 min for approximately 30 min) following a protocol in anesthetized pigs similar to the one used for LASCA. Due to the increased skin penetration depth of the LDI system, subcutaneous injections were performed at a 3 mm depth using a 32G needle. Injections were performed in areas that could be scanned in the same image. Normalized perfusion units at 6.53 min (first data point after injection) were compared using a paired t-test.

## Results

### Niacinamide Increases the Rate of Insulin Aspart Absorption in Pigs

**Fig. 1:**
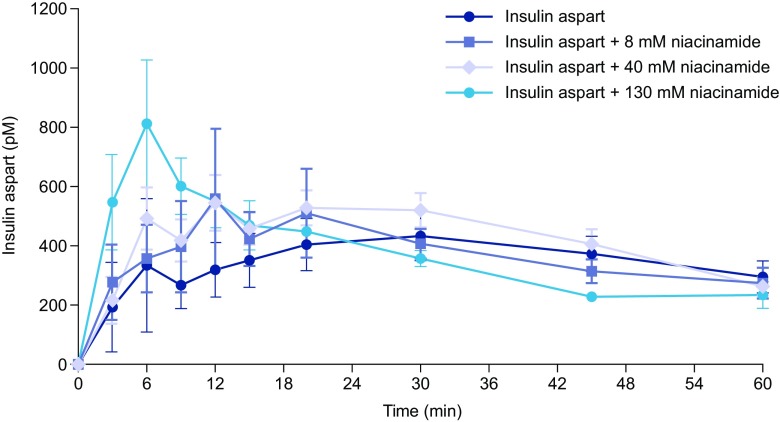
Plasma insulin aspart concentration–time profiles after subcutaneous administration of insulin aspart formulations with niacinamide (0, 8, 40 and 130 mM) in pigs. Data are mean ± standard error of the mean (SEM), n=8.

To demonstrate the impact of niacinamide on insulin absorption, dose-response studies were performed in pigs. Approximately 60% of the total AUC_0–360_ occurred during the first 60 min. Niacinamide increased the rate of early insulin absorption into the plasma in a dose-dependent manner over the 8–130 mM range (Fig. 1). The rate of absorption, measured as the AUC_0-15_/AUC_0-60_ ratio, was higher for insulin aspart formulated with niacinamide and borderline significant at the 130 mM concentration (*p*=0.07), and included data from an outlier profile in the reference group without niacinamide. Formulations with 1-methyl-niacinamide did not increase the rate of absorption of insulin aspart (data not shown), supporting its use as a tool to explore the mechanistic properties of niacinamide.

### Niacinamide Disappears Faster than 1-methyl-niacinamide from Subcutaneous Injection Depots

**Fig. 2:**
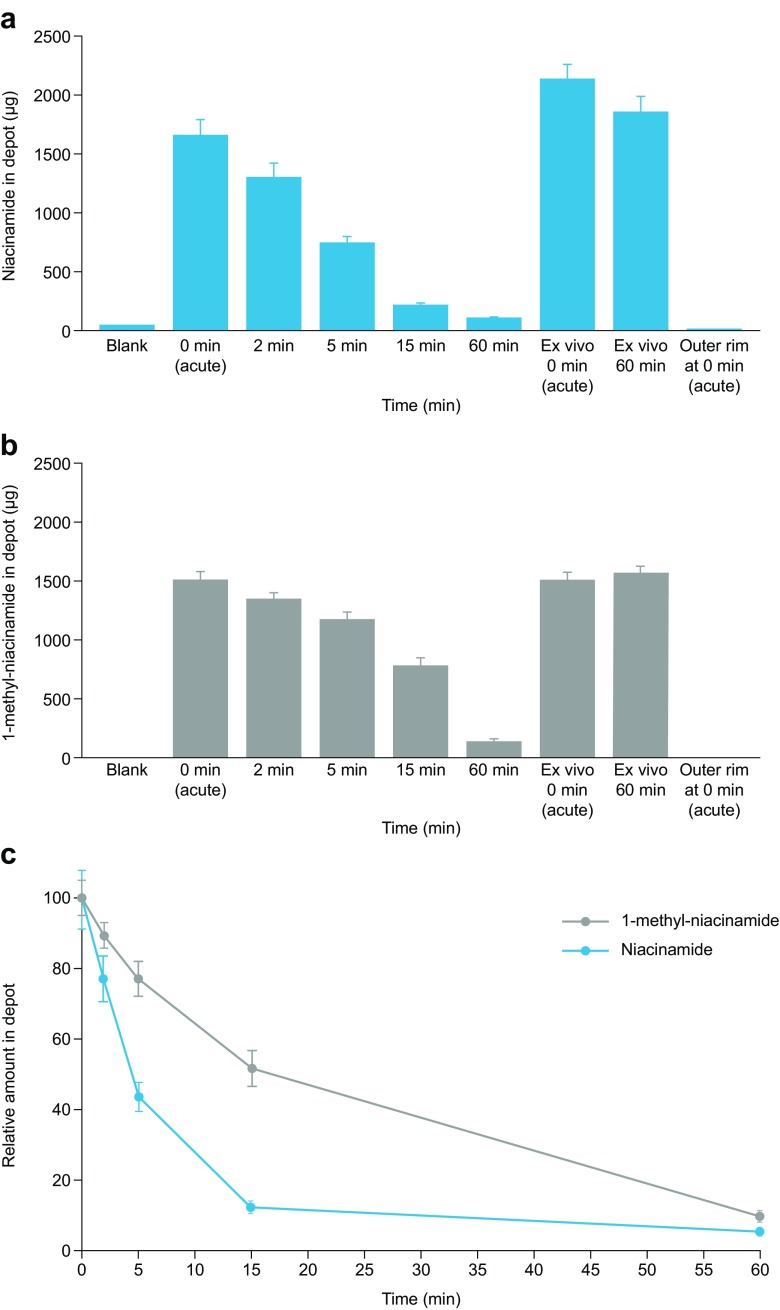
LC-MS/MS quantification (mean ± SEM) of (**a**) niacinamide (n=9) and (**b**) 1-methyl-niacinamide (n=3), and (**c**) normalized time–dependent quantification of niacinamide (n=9) and 1-methyl-niacinamide (n=3) in sample homogenates. SEM, standard error of the mean.

The rate of niacinamide disappearance was investigated in subcutis biopsies from pigs. Niacinamide disappeared faster than 1-methyl-niacinamide over the first 15 min, and niacinamide decreased to amounts comparable to the blank control by 60 min post-injection (Fig. 2a, b). The absence of detectable compounds in tissue from the outer rim around the 0 min (acute) depot shows that the injected compounds were entirely confined within the excised tissue depots, and that the disappearance likely mirrors absorption into the circulation. The amount of niacinamide in the *ex vivo* samples was similar to the amount in the 0 min (acute) sample (Fig. 2a), highlighting that an active blood supply is required for absorption.

Detection of 1-methyl-niacinamide revealed similar trends as found in the samples containing niacinamide; the amount of 1-methyl-niacinamide in *ex vivo* samples was similar to the amount quantified in the 0 min (acute) sample, and there were non-detectable amounts in the combined outer rim samples as well as in blank samples (Fig. 2b). The time-dependent disappearance of niacinamide and 1-methyl-niacinamide is illustrated in Fig. 2c. The disappearance of niacinamide from the samples was considerably faster than the disappearance of 1-methyl-niacinamide, and the half-lives were estimated (based on a fit to an exponential decay using a combined error model) to 4.8 min (90% confidence interval [CI] 4.2–5.4) and 17.2 min (90% CI 15.1–19.3), respectively.

### Niacinamide Increases the Early Absorption Rate of Insulin Aspart in Human Patients with Diabetes

**Fig. 3:**
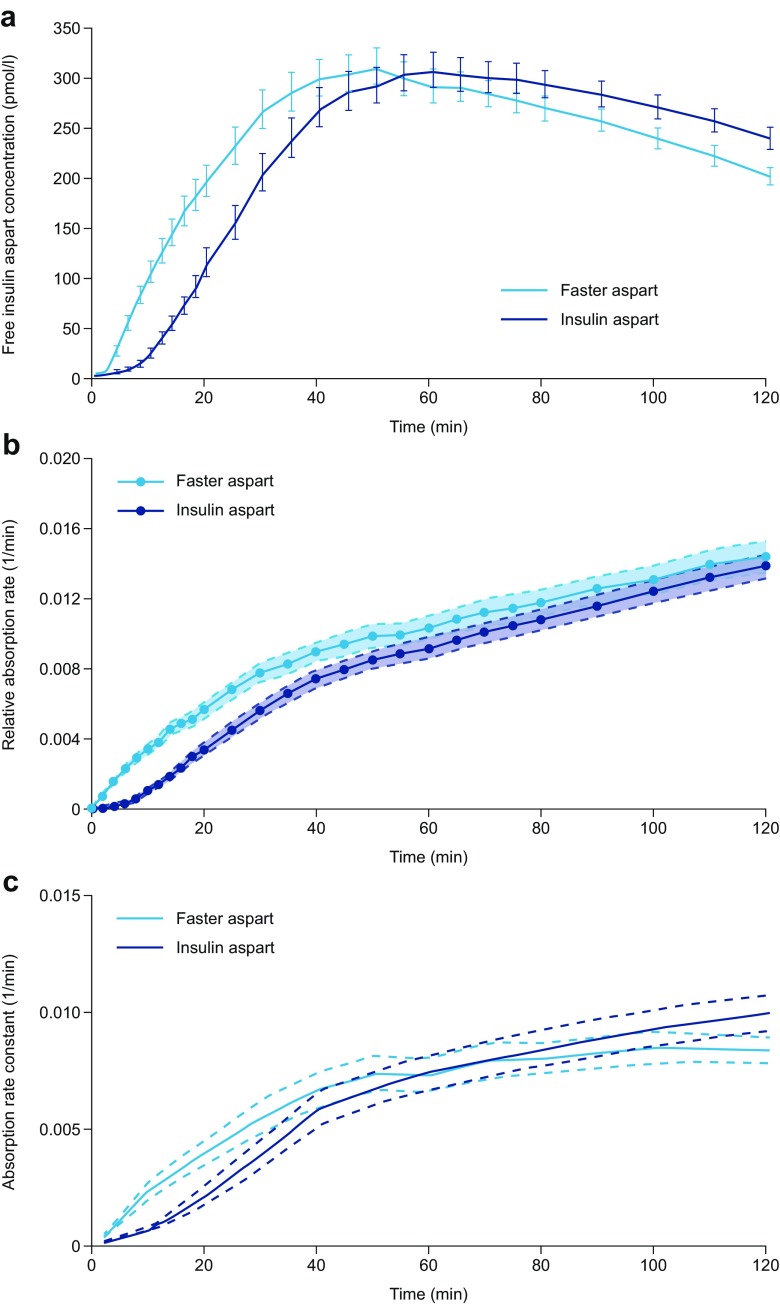
(**a**) Mean serum insulin aspart concentration–time profiles for faster aspart and insulin aspart. Patients with T1D received a single subcutaneous dose of 0.2 U/kg faster aspart or insulin aspart, and blood samples for serum insulin aspart were taken for 12 h. (**b**) Rate of absorption relative to the amount of insulin aspart in the subcutaneous depot (deconvolution approach). Lines between data points were linearly interpolated. Data shown as mean ± SEM. (**c**) Absorption rate constant profiles (population modelling approach). Data shown as geometric mean and 95% CI. Part 3A adapted from Heise *et al. Diabetes Obes Metab* 2015:17;682–688, with permission of John Wiley & Sons, © 2015. CI, confidence interval; faster aspart, fast-acting insulin aspart; SEM, standard error of the mean; T1D, type 1 diabetes.

As previously published by Heise *et al*., a left shift in the mean serum insulin aspart concentration–time profile was observed for faster aspart compared with insulin aspart in patients with T1D (Fig. 3a) (25). To demonstrate the impact of niacinamide on the early absorption of insulin aspart relative to the amount in the subcutaneous depot, absorption rates of insulin aspart were estimated using two different modelling approaches. The two approaches to translating the mean profiles in Fig. 3a into relative absorption rates yielded very similar results up to approximately 30–40 min after injection, demonstrating an effect of niacinamide on insulin aspart absorption during this time period (Fig. 3b, c). Beyond this time period, the two models differ slightly, although it is likely that the relative rate of absorption is similar between the two formulations. This is compatible with the absolute rate of absorption being reduced for faster aspart in the late phase (after ~60 min; Fig 3a) when the insulin depot is smaller due to the increased initial absorption.

### Niacinamide Increases the Monomeric Fraction of Insulin Aspart as Assessed by X-Ray Scattering

**Fig. 4:**
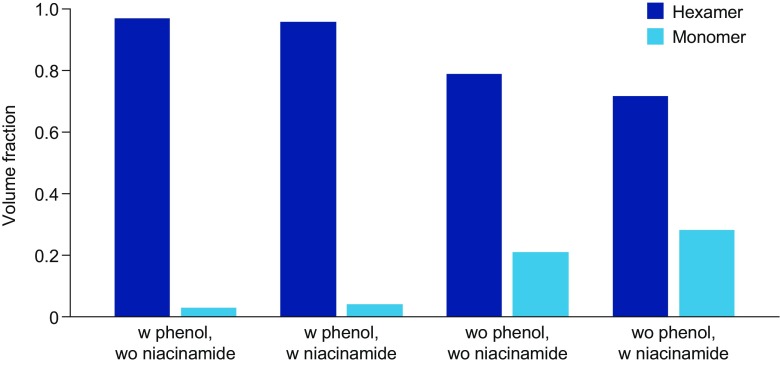
Volume fractions of insulin aspart monomers and hexamers estimated based on the recorded SAXS data. SAXS, small angle X-ray scattering; w, with; wo, without.

After subcutaneous administration, insulin aspart dissociates from hexamers, which are too large to pass easily through capillary membranes, into more easily absorbed dimers and monomers. The dissociation of insulin aspart is the rate-limiting step of insulin absorption and onset of action. The impact of niacinamide on the oligomerization state of insulin aspart under physiological relevant conditions was analyzed by SAXS. The preservative excipients phenol and m-cresol in the insulin aspart formulation are well-known to promote hexamer formation by stabilizing insulin in R6-state (29), and the effect of phenol and m-cresol are clearly reflected in the SAXS data, which show that insulin aspart is considerably more monomeric in the absence of phenol and m-cresol (Fig. 4). Contrary to the effect of phenol and m-cresol, however, niacinamide increased the monomer content. When comparing the samples pair-wise, it is evident that niacinamide gives rise to ~35% (V/V) increase in relative monomer fraction independently of the presence of phenol and m-cresol (Fig. 4).

After the dissociation of the hexamers, insulin dimers may form oligomers in the subcutaneous tissue, which also impedes absorption (30). See Supplementary Material for further SAXS data demonstrating the effect of niacinamide and 1-methyl-niacinamide on Zn^2+^-free oligomer formation under emulated subcutaneous conditions (Supplementary Fig. S2 and S3).

### Niacinamide Increases the Transport of Insulin Aspart Across an Endothelial Cell Barrier *In Vitro*

**Fig. 5:**
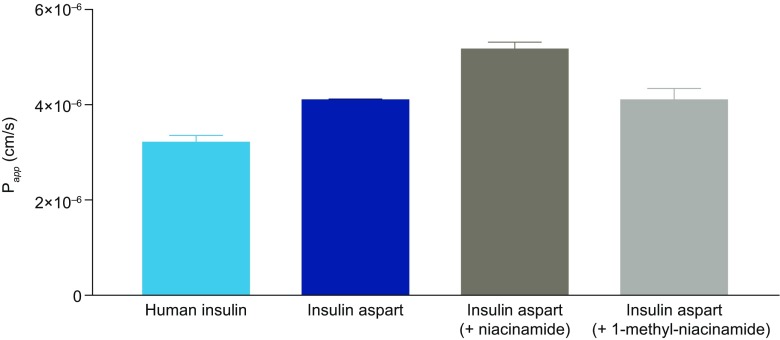
Effect of niacinamide on transport of human insulin or insulin aspart across HDMEC monolayers. Data are mean ± SEM, n=3–4. HDMEC, human dermal microvascular endothelial cell; P_app_, apparent permeability; SEM, standard error of the mean.

To evaluate the effect of niacinamide on absorption across the vascular endothelium, the permeation of insulin aspart with and without niacinamide was evaluated using HDMEC monolayers. The apparent permeability (*P*_*app*_) of insulin aspart was increased by ~27% (4.1 x 10^-6^ cm/s *vs* 5.2 x 10^-6^ cm/s, respectively) in the presence of niacinamide, and by ~63% compared with human insulin (3.2 x 10^-6^ cm/s) (Fig. 5). In contrast, 1-methyl-niacinamide did not augment the *P*_*app*_ of insulin aspart (4.1 x 10^-6^ cm/s). Additionally, a noticeable upstroke in the amount of insulin aspart accumulated in the receiver chamber over the first 15 min was observed with the formulations containing niacinamide compared with those without niacinamide. This was reflected in the ratios of insulin aspart accumulated in the first 15 min (accumulated_0-15min_/amount accumulated_0-60min_ [%]), which also revealed that niacinamide gives rise to an increase relative to either insulin aspart alone or human insulin (33.2% *vs* 24.7% *vs* 18.1%, respectively), while 1-methyl-niacinamide had no appreciable effect (Table III). The observed trend with niacinamide was sustained throughout the course of the study whereby niacinamide was observed to augment the ratio of insulin aspart accumulated over the first 30 min (accumulated_0-30min_/amount accumulated_0-60min_; 49.6% with niacinamide *vs* 43.4% for insulin aspart alone) and 45 min (accumulated_0-45min_/amount accumulated_0-60min_; 73.2% with niacinamide *vs* 65.3% for insulin aspart alone). These differences in the presence of niacinamide were reflected in the absolute amount of insulin aspart accumulated at the different time points (15, 30, 45 and 60 min) (data not shown). Comparable effects of niacinamide on insulin aspart permeation were also observed in the absence of Zn^2+^ (Supplementary Fig. S4).

**Table III:** Amount Accumulated_0-15min_/Amount Accumulated_0-60min_ (%) in Trans-Endothelial Transport Studies of Human Insulin and Insulin Aspart

Formulation	Amount accumulated_0-15min_ /amount accumulated_0-60min_ (%)
Human insulin	18.1±0.9
Insulin aspart	24.7±1.0
Insulin aspart + niacinamide	33.2±5.0
Insulin aspart + 1-methyl-niacinamide	22.4±1.4

Data are mean±SD, n=3–4. SD, standard deviation

### Niacinamide Induces Vasorelaxation of Porcine Arteries *Ex Vivo*

**Fig. 6:**
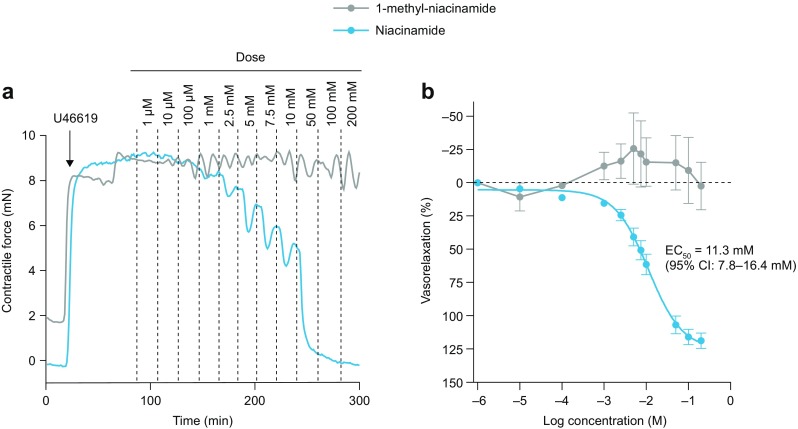
Effect of niacinamide and 1-methyl-niacinamide on vasorelaxation. Isolated porcine subcutaneous arteries were preconstricted with U46619 (1 μM) followed by stimulation with niacinamide or 1-methyl-niacinamide in cumulatively increasing concentrations. (**a**) Representative recordings of contractile force during preconstriction (arrow indicates time for addition of U46619) and stimulation with niacinamide or 1-methyl-niacinamide. (**b**) Vasorelaxation is given as a percentage between preconstricted tone (0% vasorelaxation) and baseline tone (100% vasorelaxation). Data are mean ± SEM for six animals (niacinamide) and two animals (1-methyl-niacinamide). For each animal, data from 2–4 vessel segments with each compound were averaged. CI, confidence interval; EC_50_, half-maximum effective concentration.

Cumulatively increasing niacinamide concentrations (1 μM–200 mM) induced a clear concentration-dependent vasorelaxation, with a half-maximum effective concentration (EC_50_) of 11.3 mM (95% CI 7.8–16.4 mM), whereas 1-methyl-niacinamide had no substantial effect on vascular tone (Fig. 6). A similar EC_50_ value was observed when niacinamide was added in an inverse concentration series (200 mM decreasing to 1 μM) that mimicked the dilution and disappearance of niacinamide in the subcutaneous space after an injection (data not shown). The highest concentration of niacinamide (200 mM) induced maximal vasorelaxation in all segments regardless of vessel diameter, whereas lower concentrations of niacinamide (5 and 10 mM) gave more profound vasorelaxation in vessels with a luminal diameter of 100–200 μm compared with vessels with a diameter of 600–700 μm (Supplementary Fig. S5).

### Niacinamide Increases Regional Subcutaneous Blood Flow in Pigs

Three different techniques were employed to monitor the possible *in vivo* consequence of niacinamide-mediated vasorelaxation on local blood flow and skin perfusion in pigs.

#### Xe-133 Washout Studies

**Fig. 7:**
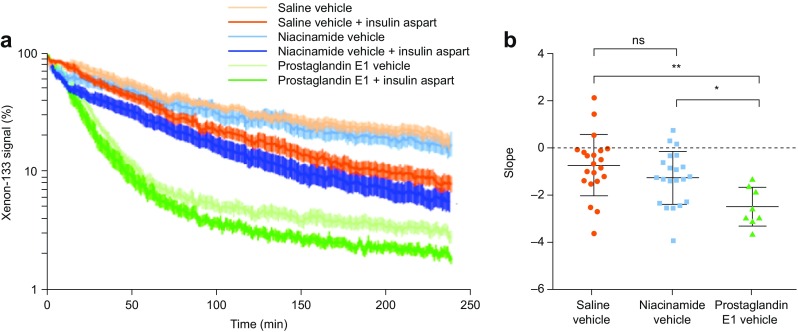
(**a**) Xenon-133 signal washout after injection to the neck of pigs. Mean ± SEM normalized to 100%; n=21 for saline, n=21 for niacinamide group and n=8 for the prostaglandin E1. (**b**) Regression line slopes for niacinamide, prostaglandin and saline control vehicles (without insulin aspart) during the initial 0–15 min interval. Mean ± SD of the regression line slopes for each group. *p≤0.05; **p≤0.01. ns, non-significant; SD, standard deviation; SEM, standard error of the mean.

Normalized Xe-133 measurements after injection of insulin aspart and niacinamide (170 mM), prostaglandin and saline vehicles into the necks of pigs are shown in Fig. 7a. The formulations containing prostaglandin show an increased washout from the injection site compared with those containing saline and niacinamide. Insulin itself acts as a vasodilator, and this can also be observed in the washout profiles. To study the effect of niacinamide on the early phase of absorption, Xe-133 washout was analyzed in the 0–15 min interval for each vehicle (without insulin aspart). The mean (± standard deviation, SD) regression line slope was –0.71% (±1.30) per min for saline, –1.24% (±1.12) per min for niacinamide and –2.47% (±0.82) per min for prostaglandin (Fig. 7b). Washout in the 0–15 min interval with prostaglandin vehicle was statistically significantly different from both saline and niacinamide vehicles (*p*<0.002 and *p*<0.05, respectively). The mean regression slope of the niacinamide vehicle was not statistically significantly different from the saline vehicle, although visual inspection of Fig. 7 suggests there may be a short-acting effect of niacinamide.

#### Laser Speckle Contrast Analysis (LASCA)

**Fig. 8:**
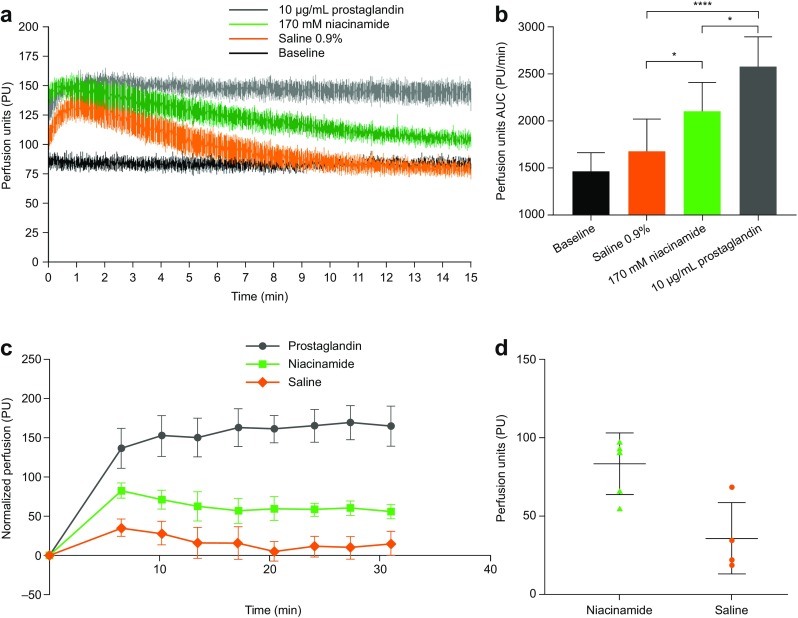
LASCA and LDI of skin perfusion in pigs after subcutaneous administration of saline, niacinamide and prostaglandin formulations. (**a**) LASCA perfusion recordings for each of the three formulations (also see supplementary video of LASCA). (**b**) LASCA AUC calculations during the 0–15 min interval. n=4; *p≤0.05; ****p≤0.00001. (**c**) Normalized LDI profiles presented as mean±SEM, n=4. (**d**) Comparison of perfusion between niacinamide and saline 6.53 min after injection presented as mean±SD, n=4; *p*<0.02. AUC, area under the curve; LASCA, laser speckle contrast analysis; LDI, Laser Doppler imaging; SD, standard deviation; SEM, standard error of the mean.

LACSA shows skin perfusion after intradermal injection of prostaglandin (10 μg/ml), niacinamide (170 mM), saline control and baseline (Fig. 8a). Analysis of the 0–15 min AUC showed statistically significant differences between saline and niacinamide (*p*=0.023), saline and prostaglandin (*p*<0.00001), and saline and niacinamide and prostaglandin (*p*=0.011). There was no statistically significant difference between saline and baseline (Fig. 8b). 1-methyl-niacinamide did not increase the LASCA signal compared with saline (see Supplementary video).

#### Laser Doppler Blood Flow Imaging (LDI)

Graphical representation of the baseline corrected LDI data illustrates that both niacinamide and prostaglandin result in increased skin perfusion compared with the saline control (Fig. 8c). By visual inspection, the prostaglandin effect appears greater and of longer duration. Paired t-test of the first data point after injection (6.53 min) revealed a statistically significant increase in skin perfusion with niacinamide compared with saline (Fig. 8d).

## Discussion

Faster aspart is a novel formulation of insulin aspart that has a faster onset of action and greater early glucose-lowering effect compared with insulin aspart (9). This study provides an investigation of the mechanism of action for the faster absorption and onset of action seen in clinical trials with faster aspart.

Studies in pigs confirmed that the excipient niacinamide concentration-dependently increases insulin aspart absorption in the mM range, similar to the concentration of niacinamide in the faster aspart formulation. No increase in absorption of insulin aspart was achieved with 1-methyl-niacinamide. The reasons for the differing effects of niacinamide and 1-methyl-niacinamide are unknown, but the molecules have been shown to differ with respect to their anti-inflammatory effects (31). Quantification of niacinamide disappearance from porcine injection depots revealed a half-life of ~5 min, which is approximately four times shorter than the half-life of 1-methyl-niacinamide. In human subjects, the addition of niacinamide resulted in a pronounced increase in the rate of insulin aspart absorption in the first ~30–40 min after injection, as demonstrated by two different approaches to analyzing the serum concentration–time profiles reported previously by Heise *et al*. (2015), coinciding with the period when niacinamide remains in the injection depot. These experiments clearly demonstrate that niacinamide mediates the acceleration of initial insulin aspart absorption; however, they do not reveal the mechanism by which this is achieved. Therefore, the influence of niacinamide on oligomerization and local blood perfusion was investigated.

The oligomerization state of insulin aspart was studied in a series of SAXS experiments designed to simulate conditions as the insulin aspart molecule transfers from the pharmaceutical formulation of faster aspart to the interstitial fluid in the subcutaneous space. Immediately after subcutaneous injection, the pharmaceutical formulation will be diluted; however, all excipients are likely to initially remain present in approximately the original ratios. By means of SAXS, it was illustrated that niacinamide increased the insulin aspart monomer fraction in diluted formulations, and this was independent of the presence of phenol and m-cresol. Within minutes of administration, depletion of excipients, such as Zn^2+^, phenol and m-cresol, in the injection depot destabilizes insulin hexamers and results in an increase in dimers and monomers. However, the presence of monovalent and divalent ions in the interstitial fluid can promote oligomerization of insulin dimers, further impeding absorption. As documented by the SAXS experiments, niacinamide has as strong counteracting effect on the Zn^2+^-independent oligomerization of insulin aspart that can occur in the presence of monovalent (Na^+^) and divalent ions (Ca^2+^ and Mg^2+^) found in the subcutaneous space (30,32).

The reduced level of insulin aspart oligomerization resulting from the presence of niacinamide likely also explains the observation of an increased permeation rate of insulin aspart in the trans-endothelial transport assays, this being especially marked in the first 15 min. The presence of niacinamide is likely to influence not only the initial distribution of oligomers in the injection depot but also the distribution throughout the entire course of the absorption process due to the hierarchical assembly of the insulin hexamer and the coupling of equilibria between the oligomers. This was reflected by a sustained increase in the amount of insulin aspart accumulated in the receiver chamber throughout the course of the transport assay with formulations containing niacinamide.

Niacinamide also induced a clear concentration-dependent vasorelaxation response in isolated subcutaneous porcine arteries, with an EC_50_ of around 12 mM, which is lower than the concentration in the faster aspart formulation. Importantly, vasorelaxation was not detected after the addition of 1-methyl-niacinamide. Analysis of each individual vessel segment revealed that smaller vessels were more sensitive to niacinamide than larger vessels. As blood flow in subcutaneous tissue is mainly regulated at the level of small resistance arteries and arterioles with luminal diameters of 100–300 μm, the findings suggest that niacinamide acts in the part of the subcutaneous vascular tree responsible for regulation of local tissue perfusion. The mechanisms underlying niacinamide-induced vasorelaxation remain unclear (33,34), although a previous study in coronary arteries suggests that niacinamide can inhibit cyclic adenosine diphosphate (cADP)-ribose formation in vascular smooth muscle cells in the mM range (19).

The impact of niacinamide-mediated vasorelaxation on skin blood flow and perfusion was studied using Xe-133 washout, LASCA and LDI methods in pigs. Xe-133 washout demonstrated a clear effect of prostaglandin E1, a known potent vasodilator, whereas no clear effect of niacinamide was observed. While the Xe-133 signal washout was not used as an absolute measurement of blood flow in this study, this method is validated to correlate with local subcutaneous blood flow (although over a longer time frame than that monitored in this study) (35). Using LASCA, a clear increase in skin perfusion with prostaglandin and an intermediate increase with niacinamide were detected compared with saline. The LASCA results were supported by the LDI analysis, where both prostaglandin and niacinamide increased skin perfusion compared with saline. There are limitations to these two techniques; LASCA technology can only detect differences close to the skin surface (~0.5–1 mm), whereas insulin is normally deposited below the skin surface (4–5 mm), while LDI penetrates deeper but has a lower temporal resolution.

## Conclusion

Faster aspart is a novel formulation of insulin aspart with a faster onset of action and early-glucose lowering action. The mechanism through which the addition of niacinamide results in faster absorption of insulin aspart into the bloodstream appears to be multifaceted. SAXS data and trans-endothelial transport assay results suggest that niacinamide increases the initial abundance of the more easily absorbable insulin aspart monomers and thereby initial transport of insulin aspart after subcutaneous administration. In addition, the measurements of vascular tension in isolated subcutaneous arteries and blood flow and skin perfusion in pigs indicate that niacinamide mediates a transient, local vasodilatory effect, which may in turn also increase absorption of insulin aspart. In combination with a rapid disappearance of niacinamide from the injection site and estimations of the duration of increased relative absorption rate being approximately 30–40 min, these investigations represent current knowledge of how niacinamide increases insulin aspart absorption in faster aspart after subcutaneous bolus injection.

## Electronic supplementary material

ESM 1 (MP4 45046 kb)

ESM 2 (PDF 797 kb)

## Data Availability

The datasets generated during and/or analyzed during the current study are available from the corresponding author on reasonable request.

## ABBREVIATIONS

ANOVA: Analysis of variance
AUC: Area under the curve
cADP: Cyclic adenosine diphosphate
CI: Confidence interval
EC_50_: Half-maximum effective concentration
EDTA: Ethylenediaminetetraacetic acid
Faster aspart: Fast-acting insulin aspart
HbA_1c_: Glycated hemoglobin
HBSS: Hank's balanced salt solution
HDMEC: Human dermal microvascular endothelial cell
HEPES: 4-(2-hydroxyethyl)-1-piperazineethanesulfonic acid
IGF: Insulin-like growth factor
ISF: Interstitial fluid-like
LASCA: Laser speckle contrast analysis
LC-MS/MS: Liquid chromatography–mass spectrometry
LDI: Laser doppler imaging
LOCI: Luminescent oxygen channeling immunoassay
LYD: Landrace-Yorkshire-Duroc
ns: Non-significant
P_app_: Apparent permeability
PBS: Phosphate buffered saline
PK: Pharmacokinetic
PPG: Postprandial glucose
PSS: Physiological saline solution
RHI: Regular human insulin
SAXS: Small angle X-ray scattering
SD: Standard deviation
SEM: Standard error of the mean
T1D: Type 1 diabetes
T2D: Type 2 diabetes
