# Supplementary material for: Elucidating the Mechanism of Absorption of Fast-Acting Insulin Aspart: The Role of Niacinamide
Source: Pharm Res. 2019 Feb 11;36(3):49. doi: 10.1007/s11095-019-2578-7 (PMC6373292; doi:10.1007/s11095-019-2578-7)
Supplement: Supplementary file 2 — (PDF 797 kb) [file 11095_2019_2578_MOESM2_ESM.pdf]

Elucidating the mechanism of absorption of fast-acting insulin aspart: the role of niacinamide

Jonas Kildegaard, Stephen T Buckley, Rasmus H Nielsen, Gro K Povlsen, Torben Seested, Ulla Ribel, Helle B Olsen, Svend Ludvigsen, Claus B Jeppesen, Hanne HF Refsgaard, Kristian M Bendtsen, Niels R Kristensen, Susanne Hostrup, Jeppe Sturis

Supplementary Material

Materials and methods

Pharmacokinetic (PK) modelling using available data from human patients with diabetes

First method for estimation of absorption rate – deconvolution approach

A two-compartment model was fitted to intravenous (i.v.) data from trial NN1218-3949 (n=20) (registered at ClinicalTrials.gov: NCT02089451) (1) using Monolix 2016R1 software (Lixoft, France). The parameters from the two-compartment model were used for an in-house MATLAB script to perform deconvolution on subcutaneous (s.c.) data for fast-acting insulin aspart (faster aspart).

The two-compartment model, with elimination from the central compartment, was parameterized as follows:

$$\frac{dn_c(t)}{dt} = a(t) - \Delta\left(c_c(t) - c_p(t)\right) - k \cdot n_p(t)$$

$$\frac{dn_p(t)}{dt} = \Delta\left(c_c(t) - c_p(t)\right)$$

$$V = Vk \cdot weight$$

$$Vc = V \cdot FC$$

$$V_p = V \cdot (1 - FC)$$

$$n_c = c_c \cdot V_c$$

$$n_p = c_p \cdot V_p$$

Where n_c is the amount in the central compartment and n_p is the amount in the peripheral compartment, c_c and c_p are the corresponding concentrations, V is the total volume of distribution, and FC is the fraction of volume in the central compartment. k is the elimination rate and V_k is the volume of distribution per kg. a(t) is the absorption rate, which is set to 0 for i.v. data.

Table I: Population parameters for insulin aspart administered intravenously

Parameter	Explanation	Population value
Δ	Inter-compartmental clearance	0.179 L/min
FC	Fraction of volume of central compartment	0.634
k	Elimination rate	0.293 1/min
Vk	Volume of distribution/kg	0.0774 L/kg

Derivation of absorption rate

For estimation of the rate of absorption a(t), the first step is to solve the second differential equation using the general solution to a first order linear differential equation:

$$y' + f(x) \cdot y - g(x) = 0$$

Which has the solution:

$$y = e^{-F(x)}\left(\int e^{F(x)} g(x)dx + C\right)$$

Where F(x) is the antiderivative of f(x). The second differential equation is:

$$\frac{dn_p(t)}{dt} = \Delta c_c(t) - \frac{\Delta}{V_p} n_p(t)$$

Identifying that F(x) is $\frac{\Delta}{V_p} \cdot t$ and g(x) is $\Delta c_c(t)$, and inserting limits:

$$n_p(t) = e^{-\frac{\Delta}{V_p}\cdot t}\left(\int_0^t e^{\frac{\Delta}{V_p}\cdot t} \Delta c_c(t)dt + C\right)$$

for $n_2(t = 0) = 0$

$$0 = e^{-\frac{\Delta}{V_p}\cdot 0}\left(\int_0^0 e^{\frac{\Delta}{V_p}0} \Delta c_1(0)dt + C\right)$$

$$0 = 1\left(\int_0^0 1\Delta c_1(0)dt + C\right)$$

$$0 = C$$

The solution is:

$$n_p(t) = e^{-\frac{\Delta}{V_p}\cdot t}\left(\int_0^t e^{\frac{\Delta}{V_p}\cdot t} \Delta c_c(t)dt\right)$$

Dividing by V_p and inserting back into the first differential equation the dependency on c_p is removed:

$$\frac{dn_c(t)}{dt} = a(t) - \Delta\left(c_c(t) - \frac{1}{V_p} e^{-\frac{\Delta}{V_p}\cdot t}\left(\int_0^t e^{\frac{\Delta}{V_p}\cdot t} \Delta c_c(t)dt\right)\right) - k \cdot n_c(t)$$

Rearranging the terms and expressing amounts as concentrations gives:

$$a(t) = V_c \frac{dc_c(t)}{dt} + \Delta\left(c_c(t) - \frac{1}{V_p} e^{-\frac{\Delta}{V_p}\cdot t}\left(\int_0^t e^{\frac{\Delta}{V_p}\cdot t} \Delta c_c(t)dt\right)\right) + k \cdot c_c(t)V_c$$

The differentiation of concentration was approximated by the discretization:

$$\frac{dc_c(t)}{dt} \approx \frac{c_c(t_2) - c_c(t_1)}{t_2 - t_1}$$

And the derivatives were padded by duplicating the last derivative.

For each time step, the absorption rate at the next timepoint a_{t2} can be calculated by knowing the parameters from i.v. (V_c,V_p,Δ,k), the current concentration and the next concentration.

Relative absorption rate

The cumulative sum of the absorption rate a_t yields the total amount of insulin entering the central compartment, which is approximated with the cumulative sum to the last datapoint:

$$Amount = \sum_0^{\infty} a_t \approx \sum_0^{N} a_t$$

The amount in the s.c. depot at time t (SC_t) is hypothesized as being the total amount entering the central compartment subtracted by the amount which has already entered:

$$SC(t) = Amount \cdot e^{-a(t)t}$$

Instead of using the analytical solution, the exponential decay from the s.c. depot was solved using MATLAB's Ordinary Differential Equation solver, where the rates were linearly interpolated. This allowed for a fast setup when testing multiple microboluses with basal infusion (not in this experimental setup). At each time step, the absorption rate relative to the amount in the s.c. depot is calculated by:

$$a_{rel,t} = \frac{a_t}{SC(t)}$$

The relative absorption rate is interpreted as the average absorption rate for a molecule.

Deconvolution data

Data from trial NN1218-3978 (n=51) (registered at ClinicalTrials.gov: NCT01618188) was used for s.c. profiles with individuals dosed with insulin aspart and faster aspart (2). The deconvolution method assumes that the concentration was 0 at time 0, and data below the lower limit of quantification were removed.

Second method for estimation of absorption rate – population modelling approach

In addition to using the deconvolution approach on individual subject profiles, the insulin aspart absorption rate following s.c. administration of faster aspart and insulin aspart was also determined using a (non-linear mixed effects) population modelling approach using NONMEM software (version 7.3; ICON Development Solutions, Ellicott City, MD, USA). The PK data used for this purpose was the i.v and s.c. cross-over data for faster aspart from trial NN1218-3949 (n=20) (1) and the s.c faster aspart and insulin aspart cross-over data from trial NN1218-3978 (n=51)(2). The same data as the first approach.

First, a two-compartment linear disposition model was fitted to the i.v. data. The model was parameterized in terms of clearances and distribution volumes (CL, V_c, Q, V_p); between-subject variability (BSV) was included on all four parameters and assumed to follow a log-normal distribution with an unstructured covariance matrix, and a proportional error model was used. The parameter estimates from this model were then used to describe the disposition when analyzing the s.c. data. The individual post hoc parameter estimates were used for analysis of the data in trial NN1218-3949 (the same subjects as for i.v.), whereas the population mean parameter estimates were used for analysis of the data in trial NN1218-3978 (not the same subjects as for i.v.).

In both cases, the model used to describe the absorption was a first-order absorption model, parameterized in terms of a bioavailability parameter (F) and a single absorption rate constant (k_A), which was then assumed to vary over time in a piece-wise linear manner using an appropriate time grid (with slope changes at 5, 10, 15, 20, 30, 40, 50, 60, 70, 80, 100, 120, 150, 180, 240 and 360 min after dosing) in order to mimic the assumption-light, non-parametric estimation of the deconvolution approach. BSV was included on F as

well as on the value of k_A for each of the points in the time grid, the latter with the additional constraint of a common BSV variance across the 16 different k_A values. Using the individual *post hoc* parameter estimates of k_A in the grid points, individual k_A profiles were determined, summarized by treatment using the geometric mean and 95% confidence interval, and plotted.

Common to both methods used to estimate the relative absorption rate is that the estimation of remaining insulin aspart in the depot becomes more and more uncertain when the depot becomes small. Since the relative rate of absorption is calculated by using the depot size in the denominator, it follows that there is a limitation in how long time after injection the calculations are reasonble. Hence, we have truncated calculations at 2 hours, at which point only approximately 25% of the injected insulin aspart remains in the depot.

X-ray scattering to assess the influence of niacinamide on insulin aspart oligomerization

SAXS data collection and analysis

Figure S1: Background-subtracted SAXS scattering profiles obtained for insulin aspart (0.3 mM) diluted in HBSS.

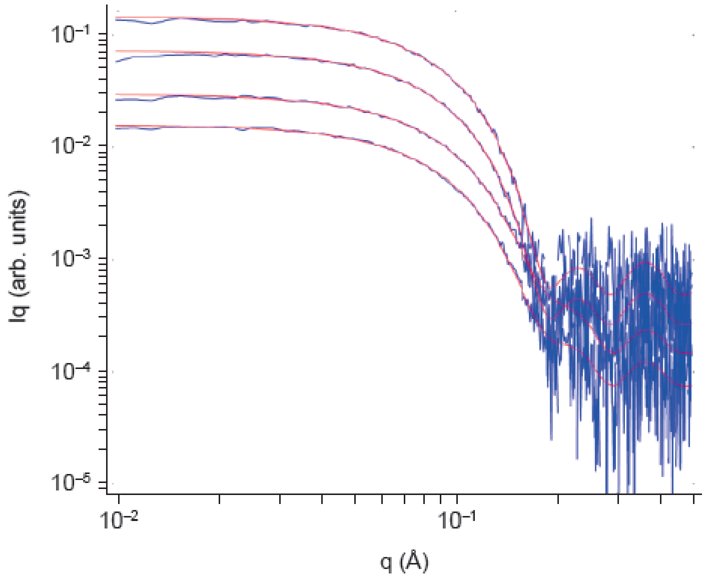

Scattering curves corresponding to sample 1 to 4 from top to bottom (see Table II in main manuscript for sample composition). arb., arbitrary; HBSS, Hank's Balanced Salt Solution; SAXS, small angle X-ray scattering.

Results

Niacinamide has a counteracting effect on the Zn^{2+}-free oligomerization of insulin aspart

After the dissociation of hexamers, monovalent (Na^+) and divalent ions (Ca^{+2}, Mg^{+2}) present in the s.c. tissue can promote the oligomerization of insulin dimers, further impeding absorption (3, 4). The impact of niacinamide on Zn^{2+}-independent oligomerization of insulin aspart was investigated using phosphate buffered saline (PBS) buffer (140 mM NaCl, 10 mM phosphate) and interstitial fluid-like (ISF) buffer (140 mM NaCl, 4 mM KCl, 2 mM $CaCl_2$, 1 mM $MgSO_4$, 10 mM phosphate). The average oligomeric number of insulin aspart was 2.7 in PBS buffer, and this increased to 5.1 in ISF buffer (Figure S2B). Hence, the additional cations in the ISF buffer appear to promote further oligomerization in addition to that caused by the physiological concentration of NaCl. With the addition of niacinamide (230 mM), the average oligomer number decreased from 2.7 to 1.4 in the PBS buffer and from 5.1 to 2.1 in the ISF buffer. Based on these observations, niacinamide may have a counteracting effect on oligomerization at the injection site, and in turn enhance the s.c. absorption rate of insulin aspart.

Figure S2. Zn^{2+}-free self-association of insulin aspart in PBS and ISF buffer. (A) SAXS data after background subtraction. The decline in the forward scattering intensity (*q*(0)→zero) in the presence of niacinamide reflects the decrease in the average molecular mass. (B) Average oligomer number calculated based on forward scattering from the SAXS data.

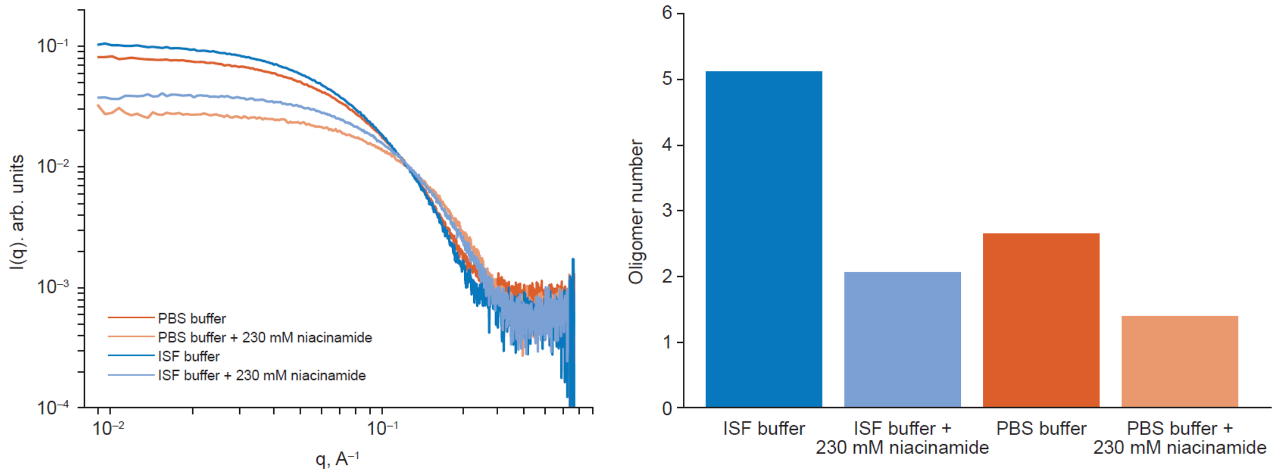

arb., arbitrary; ISF, interstitial fluid-like; PBS, phosphate buffered saline; SAXS, small angle X-ray scattering.

The effect of niacinamide and 1-methyl-niacinamide on the Zn^{2+}-free oligomerization of insulin aspart in ISF buffer was also investigated with two concentration series. The concentration of insulin aspart was kept constant at 0.6 mM, while the concentrations of the two excipients were varied from 0 to 320 mM. The effect of the two excipients was noticeably different. As can be seen from the recorded SAXS curves in Figure S3,

niacinamide had a stronger suppressing effect on oligomerization of insulin aspart compared with 1-methyl-niacinamide. Moreover, as judged by the maximal value of the P(r)-functions (maximum intramolecular distance, D_{max}), 1-methyl-niacinamide appears largely incapable of suppressing the largest oligomers. In contrast, the D_{max} values obtained for niacinamide gradually decreased as the concentration of niacinamide increased. Lastly, it should be noted that the result obtained for 1-methyl-niacinamide appears less systematic, which is seen in the plot of the radius of gyration versus concentration (Figure S3, bottom left panel). It is possible that 1-methyl-niacinamide might have a detrimental impact on the stability of insulin apart, and that this could be the underlying course for the noisier trend observed for this compound.

Figure S3. Counteracting effect of niacinamide and 1-methyl-niacinamide on Zn^{2+}-free oligomerization of insulin aspart. Left-hand side: background subtracted SAXS data showing the scattering from samples with varying concentrations of niacinamide (top) and 1-methyl-niacinamide (bottom). Middle: P(r)-functions showing the distribution of intra-particle distances in one dimension for the samples containing niacinamide (top) and 1-methyl-niacinamide (bottom). Right-hand side: calculated oligomeric number (top) and radius of gyration (bottom) versus excipient concentration.

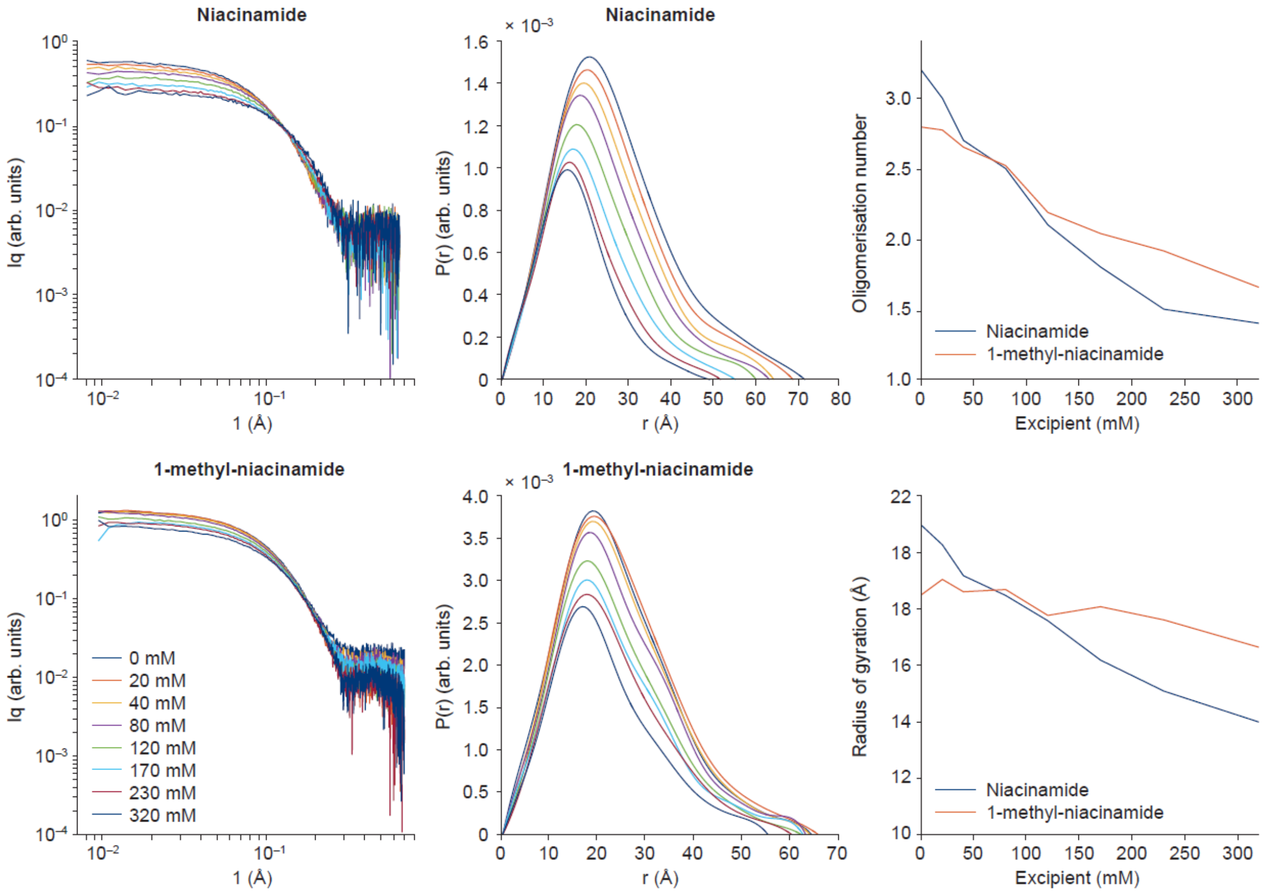

arb,. arbitrary; SAXS, small angle X-ray scattering.

Niacinamide increases Zn^{2+}-free transport of insulin aspart across an endothelial cell barrier in vitro

Figure S4. Effect of niacinamide on transport of insulin aspart across HDMEC monolayers in the absence of Zn^{2+}.

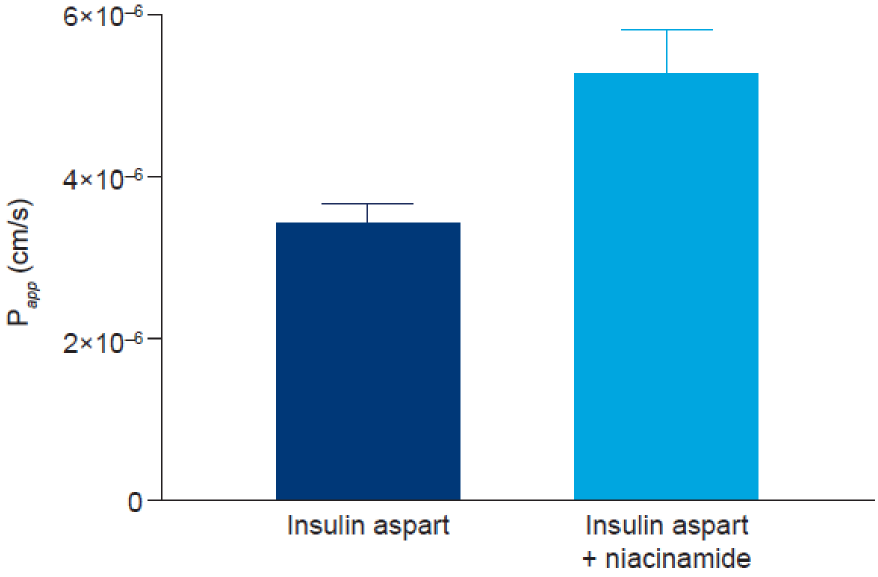

Data represents means ±SEM, n=4. HDMEC, human dermal microvascular endothelial cell; P_{app}; apparent permeability; SEM, standard error of the mean;

Niacinamide induces vasorelaxation of porcine arteries ex vivo

Figure S5: Vasorelaxation induced by different doses of niacinamide in individual vessel segments as a function of the intraluminal diameter of the individual vessel segment.

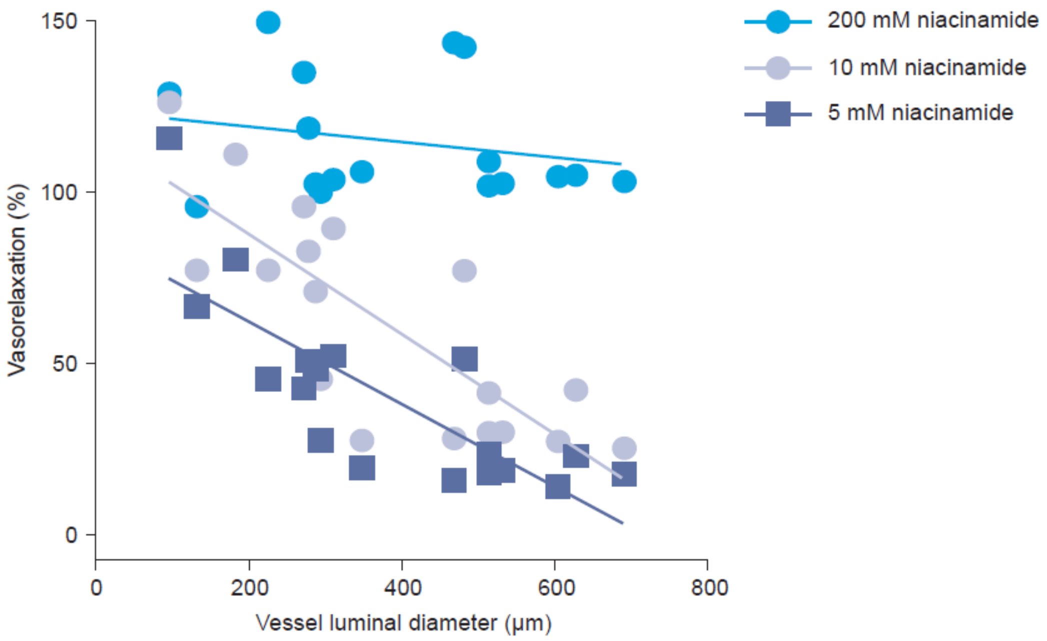

Niacinamide increases subcutaneous blood flow in pigs

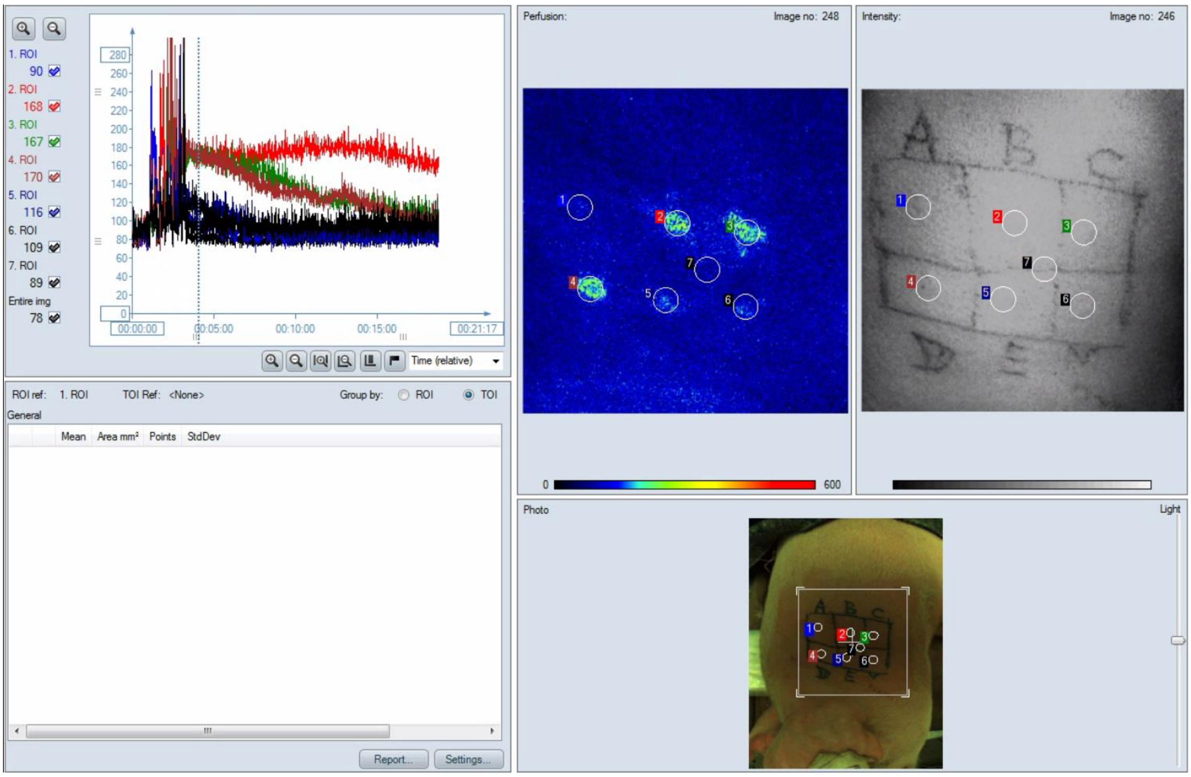

Video: Representative LASCA perfusion experiment.

LASCA images were recorded following intradermal injection of saline, prostaglandin E1 and niacinamide formulations into the neck area of anesthetized pigs. Saline (170 mM NaCl, 10 mM phosphate, pH 7.4) was injected into region 1 (blue) and 6 (black), 10 µg/ml prostaglandin E1 (170 mM NaCl, 10 mM phosphate, pH 7.4) was injected into region 2 (red), 170 mM niacinamide (85 mM NaCl, 10 mM phosphate, pH 7.4) was injected into region 3 (green), and 170 mM 1-methyl-niacinamide (10 mM phosphate, pH 7.4) was injected into region 5 (dark blue) (since 1-methyl-niacinamide is a chloride salt, no further addition of tonicity modifier was necessary). Compound x (region 4, dark red) was an additional compound studied for vasodilation but is not included in the present analysis.

References

1. Hovelmann U, Heise T, Nosek L, Sassenfeld B, Thomsen KMD, Haahr H. Pharmacokinetic properties of fast-acting insulin aspart administered in different subcutaneous injection regions. Clin Drug Investig. 2017;37(5):503–9.
2. Heise T, Hovelmann U, Brondsted L, Adrian CL, Nosek L, Haahr H. Faster-acting insulin aspart: earlier onset of appearance and greater early pharmacokinetic and pharmacodynamic effects than insulin aspart. Diabetes Obes Metab. 2015;17(7):682–8.
3. Pedersen JS, Hansen S, Bauer R. The aggregation behavior of zinc-free insulin studied by small-angle neutron scattering. Eur Biophys J. 1994;22(6):379–89.
4. Attri AK, Fernandez C, Minton AP. pH-dependent self-association of zinc-free insulin characterized by concentration-gradient static light scattering. Biophys Chem. 2010;148(1-3):28–33.
